# Miscellaneous

**Published:** 1857-01

**Authors:** 


### MISCELLANEOUS.

We wish we could respond to letters received from time to time from old subscribers with renewals, with their expressions of goodwill and encouragement. We have circulated some thousands of the *Journal* at our own expense among clergymen, when they contained articles which we thought might be useful to them. To many of them, their health is their living, and we know that by intelligent precaution, months of labor could be saved in some instances. A single hint, now and then, wisely acted on, is of incalculable value. One of them writes: "I wanted to have taken your *Journal* last year, but being a missionary on a small salary, I could not well spare the subscription price, small as it is." Just think of it, Christian people! that the day laborers, the working men of the church, its hardy pioneers, its hewers of wood and drawers of water, are so poorly paid by you, that out of a whole year's salary, not one, but many, cannot spare a dollar for instructions as to how they may keep well. It ought not so to be.

A returned foreign missionary, whom we had an opportunity of taking from the shelf and placing in active and efficient pastoral duty, says: "The *Journal* has become a sort of necessity to me; I can say of it, what I cannot of the many periodicals I receive, I read every article of every number. Ministers are so poor that they dare not subscribe for anything which is not absolutely essential to them, as men possessing minds and hearts. For the poor jaded body they can afford to do nothing. The majority of us find it impossible to keep a horse, and impossible to hire one, except on an emergency. But, although I cannot ride abroad for my health, I have learned by experience that I am none the poorer for taking the *Journal.* The hints which it contains from time to time, have often been of real service to me, and I am thankful for that kind providence which placed me, when a sufferer, under the care of its editor."

In renewing her subscription, a Southern lady writes: "I shall ever be grateful for the good you have done me, and I believe you have saved my life, probably for many years."

If by our teachings we add to the lives of those who, in the language of our fair correspondent, "could not have lived much longer without them," we may be allowed to hope that we are not living wholly in vain.

From lawyers, physicians, clergymen, eminent theologians, presidents, professors, and principals in schools, colleges and universities, and what is not less highly prized, the almost unanimous sustention of the newspaper and periodical press of the country,—from these, and a subscription list of fifty per cent. larger than we have hitherto had on a New Year's day, we cheerfully *gird our armor on* for the labor of EIGHTEEN HUNDRED AND FIFTY-SEVEN. And with the information from a correspondent, that our *Journal* is to be adopted as a reading book in a popular female seminary, we hope to write no word that is not courteous, and useful, and true.

BACK VOLUMES,

1, 2 and 3, bound, are all sold. A new supply in uniform cloth binding will soon be ready, and unfilled orders will be immediately attended to. Persons wishing to exchange their numbers for the bound volumes will be credited with seven cents for each returned number. The bound volumes are $1 25 each, and ten cents additional, if sent post-paid by mail.

